# The Impact of Endocrine-Disrupting Chemicals on Embryonic Recurrent Implantation Failure: A Narrative Review

**DOI:** 10.3390/jox16010030

**Published:** 2026-02-08

**Authors:** Anastasios Potiris, Panagiotis Antsaklis, Panagiotis Christopoulos, Nikolaos Kathopoulis, Efthalia Moustakli, Ismini Anagnostaki, Eirini Drakaki, Nefeli Arkouli, Aikaterini-Lydia Vogiatzoglou, Athanasios Zikopoulos, Sofoklis Stavros, Charalampos Theofanakis

**Affiliations:** 1 Third Department of Obstetrics and Gynecology, University General Hospital “ATTIKON”, Medical School, National and Kapodistrian University of Athens, 12462 Athens, Greece; apotiris@med.uoa.gr (A.P.); vogiatzoglou.lydia@gmail.com (A.-L.V.); sfstavrou@med.uoa.gr (S.S.); ctheofan@med.uoa.gr (C.T.); 2 First Department of Obstetrics and Gynecology, Alexandra Hospital, Medical School, National and Kapodistrian University of Athens, 11528 Athens, Greece; pantsak@med.uoa.gr (P.A.); nickatho@med.uoa.gr (N.K.); 3 Second Department of Obstetrics and Gynecology, “Aretaieion” Hospital, Medical School, National and Kapodistrian University of Athens, 11528 Athens, Greece; panchrist@med.uoa.gr; 4 Department of Nursing, School of Health Sciences, University of Ioannina, 45500 Ioannina, Greece; 5 Faculty of Medicine, Medical School, National and Kapodistrian University of Athens, 11528 Athens, Greece; isanagnostaki3@gmail.com; 6 Department of Obstetrics and Gynecology, Tzanio Hospital, 18536 Piraeus, Greece; narkoyli@windowslive.com; 7 Torbay and South Devon NHS Foundation Trust Lowes Brg, Torquay TQ2 7AA, UK; thanzik92@gmail.com

**Keywords:** endometrial receptivity, environmental exposure, reproductive toxicology, epigenetic modification, immune regulation, hormone receptor signaling, ART

## Abstract

A significant and persistent issue in assisted reproduction is recurrent implantation failure (RIF), which is often observed even after the transfer of embryos of high morphological and/or genetic quality. Accumulating data suggest that exposure to chemicals with endocrine-disrupting effects (EDCs) may be associated with adverse implantation outcomes. Many environmentally widespread substances have the potential to interfere with the regulation of the endocrine system, affecting critical mechanisms involved in implantation, such as endometrial receptivity, steroid hormone receptor signaling, immune tolerance at the maternal–fetal interface, and the epigenetic regulation of genes that are essential for successful implantation. Experimental studies have shown that exposure to EDCs can alter gene expression in the endometrium, inflammatory pathways, and the dynamics of early embryonic development, while clinical and epidemiological data have associated increased levels of EDCs in the body with lower implantation rates in assisted reproductive technology (ART) cycles. This narrative review examines the implications of these findings in reproductive medicine, summarizes recent experimental and clinical data, and highlights the molecular mechanisms linking exposure to endocrine disruptors with recurrent implantation failure. Recognizing environmental chemical exposure as a potentially modifiable risk factor may offer new perspectives for the prevention of RIF and the development of more personalized therapeutic strategies.

## 1. Introduction

The complex and tightly regulated process of embryo implantation remains one of the most significant limiting factors in the effectiveness of ART [1]. RIF continues to be a serious clinical problem, as implantation rates have largely remained unchanged despite significant advances in ovarian stimulation, embryo culture systems, genetic testing, and endometrial preparation techniques. RIF is usually defined as the failure to achieve a clinical pregnancy after repeated transfers of morphologically and/or genetically superior embryos and represents a significant proportion of unsuccessful ART cycles [2,3]. Although various factors, such as uterine pathologies, chromosomal abnormalities, thrombophilic conditions, and immunological disorders, have been associated with RIF, the etiology remains unclear in a significant proportion of cases after the usual diagnostic evaluation [4,5]. It should be noted, however, that no universally accepted definition of recurrent implantation failure exists. Definitions vary with respect to the number of failed embryo transfers required, the developmental stage of transferred embryos (cleavage-stage versus blastocyst), and the use of morphologically versus genetically euploid embryos. This clinical heterogeneity complicates the interpretation and comparison of studies investigating factors associated with implantation failure, including environmental exposures, and increases the risk of overgeneralization when extrapolating results across different patient populations.

Successful implantation during the “implantation window” requires precise temporal and spatial synchrony between a developmentally competent embryo and a receptive endometrium [6]. Endocrine signals, immunological systems, inflammatory mediators, and strictly regulated gene expression programs all work together to govern this process. The acceptance of the semi-allogeneic fetus depends on immune tolerance at the mother-fetus interface, and signaling through progesterone and estrogen is crucial for controlling the endometrium’s development, differentiation, and receptivity [7,8]. Particularly in the case of assisted reproduction, where physiological mechanisms are already altered, even minor disruptions in immunological homeostasis, hormonal balance, or receptor function can jeopardize implantation [9,10].

In recent years, environmental and lifestyle factors have drawn more attention as potential causes of infertility. EDCs are now a big worry. EDCs are a broad family of exogenous substances that can interfere with endogenous hormonal systems by imitating, blocking, or otherwise altering hormone synthesis, metabolism, transport, and signaling [11]. Common EDCs include pesticides, plasticizers, industrial chemicals, persistent organic pollutants, and several heavy metals. Widespread and chronic human exposure results from the use of consumer and medical products, contaminated drinking water, air pollution, occupational settings, and dietary intake [12].

Notably, many endocrine disruptors can exert biological effects even at low concentrations, particularly during highly sensitive developmental stages, such as gametogenesis, implantation, and early embryonic development. The lipophilic nature of some of these compounds, their resistance to biodegradation, and their ability to accumulate in reproductive tissues raise concerns about the long-term consequences of exposure to endocrine disruptors for female fertility [13]. Moreover, increasing evidence suggests that endocrine disruptors may cause epigenetic changes, including histone modifications, alterations in DNA methylation, and changes in the expression of non-coding RNAs [14,15]. These changes may persist after exposure ends, affecting reproductive outcomes and, in some cases, being transmitted to subsequent generations.

Experimental studies have shown that exposure to EDCs disrupts critical implantation processes, including signaling through steroid hormone receptors, endometrial remodeling and receptivity, angiogenesis, and immune regulation at the maternal–fetal interface [16]. At the same time, clinical and epidemiological studies have linked the overall EDC burden in the body with adverse reproductive outcomes, such as reduced implantation rates, changes in markers of endometrial receptivity, and lower pregnancy rates in assisted reproductive cycles [17]. However, the exact role of EDCs in RIF remains inadequately understood, as the available data are often inconsistent and fragmented, and limited by significant methodological shortcomings, particularly regarding the precise assessment of environmental exposure.

Despite increasing awareness of environmental factors affecting reproductive health, exposure to EDCs is rarely incorporated into the routine clinical assessment of patients with RIF. The fact that environmental exposures represent potentially modifiable risk factors makes this gap particularly significant. A more complete understanding of how EDCs interact with the biological processes governing implantation could open new avenues for preventative, counseling, and personalized approaches in reproductive care, while also contributing to the interpretation of RIF cases that would otherwise remain unexplained [18].

This narrative review aims to clarify the underlying biological mechanisms, discuss the potential implications for assisted reproduction, and synthesize the most recent experimental and clinical data regarding the relationship between endocrine-disrupting substances and recurrent implantation failure. The work integrates knowledge from reproductive biology, toxicology, and clinical practice in assisted reproduction, highlighting the importance of incorporating environmental health parameters in the assessment and management of implantation failure.

## 2. Methodology

This manuscript is a narrative review of the literature examining the relationship between exposure to endocrine-disrupting chemicals and recurrent implantation failure. A comprehensive, non-systematic literature search was conducted using PubMed, Scopus, and Web of Science, covering studies published between approximately January 2000 and December 2025.

The search was performed between October and December 2025 using combinations of keywords and controlled vocabulary terms related to endocrine disruption and implantation. Core search concepts included endocrine-disrupting chemicals, implantation, endometrial receptivity, recurrent implantation failure, and assisted reproductive technology, adapted to the syntax of each database. Representative search strings are provided in Table 1.

Clinical research pertinent to implantation biology and assisted reproduction, in vitro studies, observational human studies, and experimental animal models were all eligible. When they offered mechanical insight or contextual significance, reviews, editorials, and case reports were taken into consideration. Excluded were studies that only addressed male infertility or reproductive outcomes unrelated to implantation.

The majority of the included papers were published in English, and there was no deliberate search for non-English studies. In accordance with the narrative review design, the selection of studies was based on biological relevance to implantation-related mechanisms and possible translational significance rather than exhaustive inclusion. The reference lists of important publications were manually screened to find additional papers.

## 3. Endocrine-Disrupting Chemicals and Human Exposure

EDCs are exogenous compounds or mixtures that may disrupt normal endocrine function and may adversely affect the health of exposed organisms and their offspring. EDCs disrupt normal endocrine function, potentially resulting in adverse health effects in exposed organisms and their progeny [19]. In contrast to traditional hazardous compounds, EDCs have non-monotonic dose-response relationships, can have biological effects even at very low concentrations, and have effects that are highly dependent on exposure duration and timing [20]. These particular characteristics make risk assessment more complex and are of particular importance for reproductive processes, which are based on a finely tuned hormonal balance [21].

EDCs are a chemically diverse group of substances widely used in consumer products, industrial processes, and agricultural applications. They include plasticizers such as bisphenols and phthalates, agricultural pesticides and fungicides, persistent organic pollutants (POPs) such as polychlorinated biphenyls and dioxins, perfluoroalkyl and polyfluoroalkyl substances (PFAS), and certain heavy metals with endocrine-disrupting properties. Many of these substances are characterized by lipophilicity, resistance to environmental degradation, and bioaccumulation, resulting in persistent internal exposure even after external sources of exposure have been reduced or eliminated [22,23].

Humans are widely and often unavoidably exposed to EDCs. It occurs mainly through the ingestion of contaminated food and beverages, inhalation of polluted air and household dust, dermal absorption from personal care products, and occupational exposure [24]. Additionally, women undergoing assisted reproduction procedures may be exposed to endocrine-active chemicals present in pharmaceutical formulations, medical devices, and clinical materials. Biomonitoring investigations have shown that several EDCs are systematically detected in blood, urine, follicular fluid, and reproductive tissues, indicating the chronic nature of this exposure [25].

The ability of several EDCs to cross biological barriers and accumulate in hormone-sensitive tissues is particularly concerning. These compounds occur in the body as complex mixtures with additive or synergistic effects, despite substantial differences in their biological half-lives. In certain situations, even low-level exposure might have disproportionate biological effects during crucial time windows, such as the early stages of pregnancy and the implantation period. Moreover, accumulating evidence suggests that EDCs can induce epigenetic modifications, leading to long-term changes in gene regulation that extend beyond direct exposure [26,27].

Despite the large body of data documenting human exposure to EDCs, their role in implantation failure remains poorly understood, highlighting a significant knowledge gap in the field of reproductive toxicology. Table 2 provides an overview of the main endocrine-disrupting activities, common sources of exposure, representative EDC classes, and their relationships with reproductive tissues and implantation-related processes.

## 4. Physiology of Embryo Implantation and Endometrial Receptivity

Successful implantation requires the precise timing of a developmentally competent embryo with a hormonally receptive endometrium. It is not a passive process, but a strictly limited event that occurs during the mid-luteal phase and is based on dynamic, bidirectional communication between maternal and embryonic tissues. Because of this narrow window of time, the process is particularly vulnerable to disturbances in endocrine signaling [37,38].

Ovarian steroid hormones are the primary regulators of endometrial receptivity, with progesterone and estrogens sequentially coordinating the stages of its proliferation and differentiation. Progesterone plays a central role in the decidualization of endometrial stromal cells, a process essential for creating a functionally receptive environment, as well as for regulating vascular adaptation, epithelial remodeling, and local immune responses [39]. Steroid hormone receptors, which act as transcriptional regulators and interact with multiple signaling pathways, mediate these actions. Consequently, even minor fluctuations in hormone availability, receptor signaling, or downstream gene expression can disproportionately affect the implantation capacity [40,41].

Simultaneously, implantation requires the establishment of a specialized immune environment that promotes both tolerance towards the semi-allogeneic embryo and the maintenance of controlled inflammatory activity [42]. The natural killer cells of the uterus, macrophages, and regulatory T lymphocytes are key immune cell populations that support angiogenesis, trophoblast invasion, and the remodeling of endometrial tissues. The intense endocrine sensitivity of the implantation process is reflected in the strict hormonal control of this immune balance, as well as in its dynamic response to local microenvironmental stimuli [43,44,45].

Implantation is determined at the molecular level by coordinated programs of gene expression that regulate cell adhesion, extracellular matrix remodeling, angiogenesis, and cellular differentiation [46]. The epigenetic control of these programs forms the basis of the endometrium’s ability to respond rapidly to hormonal stimuli while maintaining a high degree of functional plasticity. Over time, the implantation process may become more vulnerable as hormonal, metabolic, and environmental influences are incorporated through epigenetic mechanisms [47].

Overall, implantation is a strictly regulated and time-limited biological process with limited tolerance to disturbances. This inherent sensitivity provides the biological basis for understanding how exogenous chemicals with endocrine-disrupting activity can affect implantation success and increase the risk of recurrent implantation failure [30,48]. The main molecular mechanisms that support endometrial receptivity and embryo implantation are summarized in Table 3.

## 5. Endocrine Disruption and RIF

The molecular mechanisms that regulate embryonic implantation are inherently sensitive to immunological, endocrine, and epigenetic control. Many of the pathways associated with RIF directly overlap with those affected by chemicals with endocrine-disrupting activity, suggesting mechanistic convergence rather than a coincidental association [15,16]. Strong evidence that exposure to EDCs can disrupt implantation-related processes, such as endometrial responsiveness, steroid hormone signaling, immunological tolerance, and epigenetic regulation, is provided by experimental models and in vitro investigations [30,31,57].

**Figure 1:**
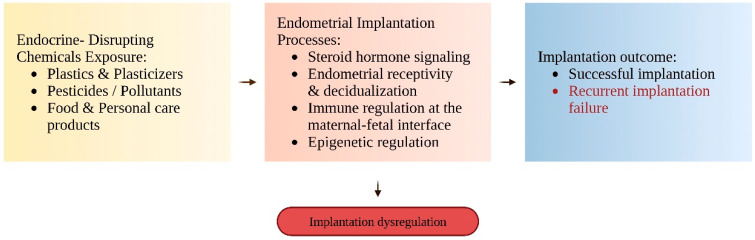
Conceptual model illustrating how exposure to EDCs may interfere with key endometrial implantation-related processes, including steroid hormone signaling, endometrial receptivity and decidualization, immune regulation at the maternal–fetal interface, and epigenetic control. Disruption of these pathways may impair implantation competence and contribute to RIF. Created in https://BioRender.com.

In contrast, human clinical evidence is more limited and predominantly derived from heterogeneous assisted reproduction cohorts, in which implantation rates, pregnancy outcomes, or overall ART successes are assessed rather than strictly defined RIF populations. Therefore, rather than straightforward causal inference in RIF, present human data mostly support associative linkages and biological plausibility [57]. Figure 1 schematically presents the key endocrine-sensitive mechanisms involved in the implantation process, as well as how chemicals with endocrine-disrupting activity can disrupt these processes.

### 5.1. Disruption of Endometrial Receptivity and Decidualization

The decidualization of endometrial stromal cells and the tightly regulated progesterone signaling are essential prerequisites for establishing endometrial receptivity. Many chemicals with endocrine-disrupting activity can interfere with downstream transcriptional activity, the expression of the progesterone receptor, or the effectiveness of its signaling, thereby limiting the endometrium’s ability to transition to a receptive state [58,59]. In experimental models, exposure to endocrine-disrupting chemicals has been associated with abnormal endometrial maturation, changes in the expression of genes related to its receptivity, and disrupted communication between stromal and epithelial cells. Such disturbances may reduce or shift the ‘implantation window,’ particularly in the context of ART, where endometrial timing is already altered, thereby contributing to recurrent implantation failure [60,61,62].

### 5.2. Alteration of Steroid Hormone Signaling Pathways

EDCs can mimic, compete with, or otherwise interfere with the normal functions of endogenous steroids, either through direct interaction with estrogen and progesterone receptors or by altering the synthesis and metabolism of steroid hormones [63,64]. In this context, non-monotonic dose–response relationships become particularly important, as low-level exposures may cause biologically significant effects without obvious toxicity [65]. Disruptions in signaling through steroid hormone receptors can lead to asynchronous endometrial maturation, abnormal patterns of gene expression, and inadequate synchronization between embryo and endometrium, features that are often observed in cases of repeated implantation failure [66]. Experimental research, for instance, has demonstrated that bisphenol A can change the expression of progesterone receptors and downstream decidualization-related gene transcription in endometrial stromal cells, offering a representative molecular mechanism by which endocrine-disrupting substances can hinder implantation.

### 5.3. Immune Dysregulation at the Maternal–Fetal Interface

Successful implantation requires a finely tuned immune environment that allows controlled inflammatory activity while simultaneously promoting immune tolerance toward the embryo. Accumulating data suggest that exposure to EDCs can affect the function of immune cells, cytokine secretion, and inflammatory signaling in reproductive organs [67]. Both excessive inflammation and insufficient immune activation, conditions resulting from a disruption of the required immune balance, are incompatible with the implantation process. Changes in immune homeostasis caused by EDCs may therefore represent a significant but underestimated environmental risk factor, given that immune dysfunction has been associated with repeated implantation failure in a substantial proportion of patients [67,68].

### 5.4. Epigenetic Modifications and Persistence of Effects

Epigenetic processes play a major role in controlling endometrial gene expression and cellular plasticity during implantation. According to experimental data, EDCs can cause epigenetic modifications, including abnormal DNA methylation patterns, changes in non-coding RNA expression, and histone modifications [47,69,70]. Even if the exposure is stopped, these alterations may continue to impact the endometrial response in subsequent cycles. In the context of RIF, where failure recurs despite changes in treatment regimens or increases in embryo quality, the permanence of such epigenetic changes becomes especially important, indicating the presence of a stable, underlying endometrial malfunction [3,71,72]. The principal pathways through which EDCs may influence the implantation process and contribute to recurrent implantation failure are outlined in Table 4.

### 5.5. Relevance to RIF

The pathways that are critical for endometrial receptivity and successful implantation are points of convergence for the mechanistic effects of chemicals with EDCs. These substances appear to act mainly through cumulative and environmentally dependent effects, weakening the functional resilience of the implantation process, rather than through a single dominant mechanism [82]. Chronic or repeated exposure to EDCs can exacerbate mild but clinically significant disturbances of endometrial receptivity, immune tolerance, or epigenetic regulation in vulnerable individuals, particularly in women undergoing ART procedures. Under these conditions, even the transfer of high-quality embryos may result in repeated implantation failure [21,83].

## 6. Clinical and Translational Implications in Assisted Reproduction

The etiology of the multifactorial condition known as RIF often cannot be adequately determined using established clinical diagnostic tools. According to an increasing amount of research data, a subset of patients, particularly those undergoing assisted reproductive treatments, may experience implantation failure as a result of environmental factors, specifically exposure to substances with endocrine-disrupting activity, which interact with critical implantation pathways [3,84,85]. It is crucial to stress, nonetheless, that direct causative links between human exposure to EDC and RIF have not yet been proven, and the majority of the evidence that is now available comes from mechanistic laboratory models and observational human investigations. Therefore, the observed convergence of reproductive and toxicological mechanisms should be taken as supporting biological plausibility rather than proving causation [86,87].

At present, the assessment of environmental exposure is not systematically included in fertility evaluation, and, currently, there are no established standards for assessing the total exposure burden to EDCs in patients with RIF [25,88]. However, association findings indicate that even low-level exposures may be associated with less favorable treatment outcomes due to the persistence and cumulative nature of many endocrine disruptors, as well as the limited physiological tolerance of the implantation process to endocrine disturbances. This factor might be especially important in the context of assisted reproduction, as repeated treatment cycles and supraphysiological hormonal stimulation may increase underlying biological susceptibility without suggesting a clear cause-and-effect relationship [23,89].

From the perspective of translational medicine, preventive and supportive strategies require a more detailed understanding of the environmental factors that affect the implantation process, as well as the development of complementary diagnostic interventions [90]. A low-risk and pragmatic addition to the modern management of RIF could include patient counseling aimed at reducing avoidable environmental exposures related to diet, occupational settings, and consumer products. In practical terms, this may involve raising awareness about common exposure sources such as frequent use of plastic food containers or medical devices, occupational contact with pesticides or industrial chemicals, and regular use of personal care products containing endocrine-active compounds. Such interventions should be viewed as precautionary and supportive rather than therapeutic and implemented with careful communication regarding the current limits of causal evidence [91,92].

Interdisciplinary collaboration, combined with the production of reliable and high-quality data, is crucial for the meaningful integration of environmental health factors into reproductive medicine [93,94]. To determine clinically significant exposure thresholds and identify vulnerable populations, prospective studies are needed that combine quantitative assessment of environmental exposure with outcomes specifically related to implantation in the context of assisted reproduction. Until such evidence becomes available, exposure to EDCs should be considered, within the framework of personalized reproductive care, a potentially modifiable associative factor rather than a confirmed causal determinant of implantation failure [86,95].

It should be explicitly acknowledged that the majority of available human evidence linking exposure to EDCs with adverse implantation outcomes is derived from heterogeneous assisted reproduction cohorts rather than from populations meeting strict diagnostic criteria for recurrent implantation failure. Many clinical studies evaluate clinical pregnancy, implantation rates, or overall ART success without differentiating between patients who experience random or first-cycle implantation failure and those who experience recurring implantation failure. Consequently, direct evidence specifically addressing well-defined RIF populations remains limited. This distinction is crucial for determining the therapeutic significance of current findings and emphasizes the necessity of future research concentrating on RIF cohorts with precise characteristics. An overview of the available human clinical and epidemiological evidence, highlighting the heterogeneity and limitations of existing studies, is provided in Table 5.

## 7. Knowledge Gaps and Future Directions

Despite the growing interest in the environmental determinants of reproductive health, significant knowledge gaps remain regarding the mechanisms through which endocrine disruptors contribute to recurrent implantation failure [11,87]. A key limitation is the lack of established and standardized methodologies for assessing environmental exposure in reproductive contexts. The majority of available data is based on single-time-point measurements, which may not capture cumulative exposure during the critical implantation window, the effects of complex chemical mixtures, or tissue-level bioavailability [96,97].

The insufficient correlation between endometrial assessment and clinically significant outcomes directly related to implantation represents another important knowledge gap [98]. Furthermore, relatively few studies focus on markers and outcomes specifically concerning the implantation process, as opposed to overall pregnancy outcomes or live birth, while even fewer specifically examine populations with repeated implantation failure. This imbalance complicates the clear distinction between primary implantation disorders and secondary pregnancy outcomes [99,100].

Further research is required to clarify whether, and to what extent, long-term exposure to low doses of complex mixtures of endocrine disruptors is associated with alterations in endometrial function throughout assisted reproduction cycles [11,25,101]. The duration and reversibility of the epigenetic changes caused by these exposures remain unclear, particularly in the context of repeated therapeutic interventions. Although experimental data suggest that certain epigenetic marks may be modifiable over time, evidence supporting reversal through lifestyle modification or therapeutic interventions in humans remains limited and largely indirect. A central goal of future research is the identification of reliable biomarkers that reflect functional impairment of endometrial receptivity, rather than merely environmental exposure [102,103].

The progress of the field is expected to depend largely on interdisciplinary approaches that bridge environmental toxicology, basic reproductive biology, and the clinical practice of assisted reproduction [104]. Future studies should integrate comprehensive morphological and functional characterization of the endometrium, assessment of repeated environmental exposures, and outcomes specifically related to implantation, aiming to clarify mechanistic pathways and strengthen causal inference while identifying vulnerable populations. At the same time, the development of translational and mechanistic frameworks that will allow the application of evidence-based practices in reproductive medicine is encouraged, going beyond the merely descriptive associations that have dominated the field to date [6,105].

## 8. Conclusions

RIF remains a complex and poorly understood clinical issue in ART. The evidence presented in this narrative review suggests that substances with endocrine-disrupting activity are capable of interacting with key biological processes involved in implantation, including immune regulation, hormonal signaling, and the epigenetic control of uterine function. While experimental models provide compelling mechanistic insights, direct causal relationships between endocrine-disrupting chemical exposure and RIF in humans have not yet been established. Current human data are therefore best interpreted as associative, supporting biological plausibility rather than definitive causation.

Given the intrinsic sensitivity of the implantation process to endocrine signaling and the widespread, often chronic nature of environmental exposure, even modest endocrine perturbations may be associated with reduced implantation efficiency, particularly in the setting of assisted reproduction. These factors are particularly important in ART, as repeated treatment cycles and hormonal manipulation may increase underlying biological vulnerability without suggesting a clear cause-and-effect relationship.

For the meaningful advancement of the field, environmental health indicators should be systematically incorporated into both clinical practice and reproductive research. Well-designed prospective studies, which combine the assessment of environmental exposure with outcomes specifically related to implantation and mechanistic investigations, are necessary to enable the transition from simple correlations to clinically and therapeutically useful applications. Ultimately, incorporating environmental factors into a broader, personalized framework of reproductive care may enhance the understanding and management of recurrent implantation failure while remaining firmly grounded in evidence-based medicine.

## Figures and Tables

**Table 1:** Summary of the literature search strategy, including databases searched, representative search terms, time frame, and last search date, used for this narrative review.

Database	Search Terms (Example)	Search Period	Last Search
PubMed	(“endocrine disrupting chemicals” OR EDCs OR bisphenol* OR phthalate*) AND (implantation OR endometrial receptivity OR recurrent implantation failure)	2000–2025	December 2025
Scopus	TITLE-ABS-KEY (“endocrine disrupt*” AND implantation AND ART)	2000–2025	December 2025
Web of Science	TS = (endocrine disrupt* AND implantation AND assisted reproduction)	2000–2025	December 2025

The asterisk (*) was used as a truncation symbol to retrieve all word variants sharing the same root (e.g., bisphenol retrieves bisphenol, bisphenols; phthalate retrieves phthalate, phthalates).

**Table 2:** The table lists the main endocrine-disrupting activities, common exposure sources, typical EDC classes, and how they relate to reproductive tissues and implantation-related processes.

EDC Class	Representative Compounds	Common Sources of Exposure	Endocrine Activity	Relevance to Reproductive Tissues/Implantation
Plasticizers (bisphenols) [28,29]	BPA, BPS, BPF	Food and beverage containers, thermal paper, medical devices	Estrogenic, anti-androgenic	Detected in serum, urine, follicular fluid; associated with altered steroid signaling and endometrial gene expression
Plasticizers (phthalates) [30,31]	DEHP, DBP, BBP	Food packaging, personal care products, medical tubing	Anti-androgenic, steroidogenic disruption	Accumulate in reproductive tissues; linked to altered hormone levels and impaired endometrial receptivity
POPs [23,32]	PCBs, dioxins	Contaminated food (especially animal fat), environmental pollution	Estrogenic, anti-estrogenic, AhR-mediated	Long biological half-lives; associated with immune dysregulation and implantation-related disturbances
Pesticides and fungicides [30,32]	DDT/DDE, atrazine, vinclozolin	Agricultural exposure, contaminated food and water	Estrogenic, anti-androgenic, thyroid-disrupting	Implicated in hormonal imbalance and altered endometrial differentiation in experimental models
PFAS [33,34]	PFOA, PFOS	Non-stick cookware, food packaging, contaminated water	Steroidogenic and thyroid interference	Detected in blood and reproductive tissues; associated with reduced fertility and implantation outcomes
Heavy metals with endocrine activity [35,36]	Cadmium, lead, mercury	Occupational exposure, smoking, contaminated food and water	Estrogen-mimetic, oxidative stress induction	Accumulate in reproductive organs; may impair hormone signaling and induce epigenetic changes

Abbreviations: endocrine-disrupting chemicals; BPA, bisphenol A; BPS, bisphenol S; BPF, bisphenol F; DEHP, di(2-ethylhexyl) phthalate; DBP, dibutyl phthalate; BBP, butyl benzyl phthalate; POPs, persistent organic pollutants; PCBs, polychlorinated biphenyls; AhR, aryl hydrocarbon receptor; DDT, dichlorodiphenyltrichloroethane; DDE, dichlorodiphenyldichloroethylene; PFAS, per- and polyfluoroalkyl substances; PFOA, perfluorooctanoic acid; PFOS, perfluorooctane sulfonate.

**Table 3:** The table outlines key regulatory mechanisms underlying embryo implantation and their association with endometrial receptivity and implantation competence.

Biological Process	Key Features	Primary Regulatory Factors	Relevance to Implantation Competence
Temporal coordination of implantation [38,49]	Restricted mid-luteal window; embryo–endometrium synchrony	Estrogen–progesterone balance	Narrow tolerance for timing errors limits implantation success
Endometrial steroid hormone responsiveness [50]	Cyclic proliferation and differentiation	Estrogen and progesterone receptors	Small alterations in signaling may disrupt receptivity
Decidualization of stromal cells [51]	Stromal differentiation; epithelial–stromal crosstalk	Progesterone signaling, transcriptional co-regulators	Essential for stable embryo attachment and invasion
Immune tolerance at the maternal–fetal interface [52,53]	Controlled inflammation; immune acceptance of embryo	Uterine NK cells, macrophages, regulatory T cells	Imbalance may impair trophoblast invasion and implantation
Angiogenesis and vascular remodeling [54,55]	Increased permeability; tissue remodeling	Hormonal and immune-mediated signals	Supports implantation and early placental development
Molecular regulation of receptivity [6]	Coordinated gene expression programs	Transcriptional and epigenetic control	Governs adhesion, invasion, and tissue plasticity
Epigenetic regulation [56]	DNA methylation, histone modification, non-coding RNAs	Hormonal and environmental inputs	Integrates signals over time; may confer persistent effects

**Table 4:** The table outlines key implantation-related pathways influenced by EDCs and their relevance to RIF.

Implantation-Related Pathway	EDCs-Mediated Effects	Biological Consequence	Relevance to RIF
Endometrial receptivity and decidualization [73,74]	Altered progesterone receptor signaling; impaired stromal differentiation	Disrupted endometrial maturation; shifted window of implantation	Reduced probability of successful implantation across cycles
Steroid hormone signaling [75,76]	Estrogenic or anti-estrogenic activity; interference with hormone synthesis and metabolism	Asynchronous endometrial development; altered gene expression	Impaired embryo–endometrium synchrony
Immune regulation at the maternal–fetal interface [77,78,79]	Modulation of cytokine profiles; altered immune cell activity	Excessive or insufficient inflammatory response	Compromised embryo tolerance and invasion
Epigenetic regulation of endometrial function [80]	Changes in DNA methylation, histone modification, non-coding RNA expression	Persistent alterations in gene regulation	Recurrent implantation failure despite changes in embryo quality
Cumulative and mixture effects [81]	Chronic low-dose exposure; additive or synergistic actions	Reduced resilience of implantation processes	Amplification of subtle defects in susceptible individuals

Abbreviations: EDCs, endocrine-disrupting chemicals; RIF, recurrent implantation failure.

**Table 5:** Summary of human clinical and epidemiological studies evaluating associations between exposure to EDCs and implantation-related or assisted reproduction outcomes. Evidence is predominantly associative and largely derived from heterogeneous ART or population-based cohorts rather than strictly defined RIF populations.

EDC Class	Representative Compounds	Study Population	Implantation-Related Outcomes Assessed	Summary of Human Evidence	Key Limitations
Bisphenols [29,57]	BPA, BPS	General ART cohorts	Implantation rate, clinical pregnancy	Repeated associations with reduced implantation or ART success	Mostly observational; limited RIF-specific data
Phthalates [25,30]	DEHP, DBP	ART and infertile populations	Implantation rate, embryo quality	Inconsistent associations across studies	Exposure assessment variability; confounding
PFAS [33,34]	PFOA, PFOS	Population-based and ART cohorts	Fertility outcomes, implantation	Emerging associative evidence	Limited implantation-specific endpoints
POPs [23,32]	PCBs, dioxins	General population	Pregnancy and fertility outcomes	Suggestive but heterogeneous associations	Long half-lives; mixed exposure profiles
Pesticides [30,82]	DDT/DDE, atrazine	Occupational and population studies	Fertility and pregnancy outcomes	Fragmented evidence	Few ART-focused studies
Heavy metals [35,36]	Cadmium, lead	Population-based cohorts	Fertility parameters	Limited and indirect evidence	Implantation rarely assessed directly

## Data Availability

No new data were created or analyzed in this study.

## Abbreviations

The following abbreviations are used in this manuscript:

EDCs	Endocrine-disrupting chemicals
RIF	Recurrent implantation failure
ART	Assisted reproductive technology
POPs	Persistent organic pollutants
PFASs	Polyfluoroalkyl substances
BPA	Bisphenol A
BPS	Bisphenol S
BPF	Bisphenol F
DEHP	Di(2-ethylhexyl) phthalate
DBP	Dibutyl phthalate
BBP	Butyl benzyl phthalate
PCBs	Polychlorinated biphenyls
AhR	Aryl hydrocarbon receptor
DDT	diclorodiphenyldicloroethylene
PFAs	Per-and polyfluoroalkyl substances
PFOA	Perfluorooctanoic acid
PFOS	Perfluoroctane sulfonate
NK	Natural killer
